# The pleiotropic roles of circular and long noncoding RNAs in cutaneous melanoma

**DOI:** 10.1002/1878-0261.13034

**Published:** 2021-06-18

**Authors:** Barbara Montico, Giorgio Giurato, Giovanni Pecoraro, Annamaria Salvati, Alessia Covre, Francesca Colizzi, Agostino Steffan, Alessandro Weisz, Michele Maio, Luca Sigalotti, Elisabetta Fratta

**Affiliations:** ^1^ Immunopathology and Cancer Biomarkers Centro di Riferimento Oncologico di Aviano (CRO) IRCCS Aviano Italy; ^2^ Laboratory of Molecular Medicine and Genomics Department of Medicine, Surgery and Dentistry 'Scuola Medica Salernitana' University of Salerno Baronissi Italy; ^3^ Genome Research Center for Health – CRGS University of Salerno Campus of Medicine Baronissi Italy; ^4^ Center for Immuno‐Oncology University Hospital of Siena Italy; ^5^ University of Siena Italy; ^6^ NIBIT Foundation Onlus Siena Italy; ^7^ Oncogenetics and Functional Oncogenomics Unit Centro di Riferimento Oncologico di Aviano (CRO) IRCCS Aviano Italy

**Keywords:** circular RNAs, cutaneous melanoma, immunotherapy, long noncoding RNAs, targeted therapy

## Abstract

Cutaneous melanoma (CM) is a very aggressive disease, often characterized by unresponsiveness to conventional therapies and high mortality rates worldwide. The identification of the activating *BRAF^V600^* mutations in approximately 50% of CM patients has recently fueled the development of novel small‐molecule inhibitors that specifically target *BRAF^V600^*‐mutant CM. In addition, a major progress in CM treatment has been made by monoclonal antibodies that regulate the immune checkpoint inhibitors. However, although target‐based therapies and immunotherapeutic strategies have yielded promising results, CM treatment remains a major challenge. In the last decade, accumulating evidence points to the aberrant expression of different types of noncoding RNAs (ncRNAs) in CM. While studies on microRNAs have grown exponentially leading to significant insights on CM biology, the role of circular RNAs (circRNAs) and long noncoding RNAs (lncRNAs) in this tumor is less understood, and much remains to be discovered. Here, we summarize and critically review the available evidence on the molecular functions of circRNAs and lncRNAs in *BRAF^V600^*‐mutant CM and CM immunogenicity, providing recent updates on their functional role in targeted therapy and immunotherapy resistance. In addition, we also include an evaluation of several algorithms and databases for prediction and validation of circRNA and lncRNA functional interactions.

## Graphical abstract

In the last decade, accumulating evidence points to the aberrant expression of different types of noncoding RNAs in cutaneous melanoma (CM). Here, we summarize and critically review the available evidence on the molecular functions of circular RNAs (circRNAs) and long noncoding RNAs (lncRNAs) in *BRAF^V600^*‐mutant CM and CM immunogenicity, providing recent updates on their functional role in targeted therapy and immunotherapy resistance. In addition, we also include an evaluation of several algorithms and databases for prediction and validation of circRNA and lncRNA functional interactions.

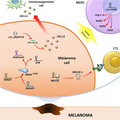

**Abbreviations**
ANRIL: antisense noncoding RNA in the INK4 locus
ASO: antisense oligonucleotide
ATB: activated by TGF‐beta
BANCR: BRAF‐activated nonprotein‐coding RNA
BRAFi: BRAF inhibitors
BSJ: back‐spliced junction
CDR1as: cerebellar degeneration‐associated protein 1 antisense transcript
CeRNA: competitive endogenous RNA
CircRNA: circular RNA
CiRNA: circular intronic RNA
CM: cutaneous melanoma
CTL: cytotoxic T lymphocyte
DIRC3: disrupted in renal carcinoma 3
DLBCL: diffuse large B-cell lymphoma
EcircRNA: exonic circular RNA
EIciRNA: exon and intron-containing circular RNA
EMT: epithelial-to-mesenchymal transition
FENDRR: FOXF1 adjacent noncoding developmental regulatory RNA
FLOT2: flotillin 2
FOXD3-AS1: FOXD3 adjacent opposite strand RNA 1
GAS5: growth arrest specific 5
GAS6-AS2: GAS6 antisense RNA 2
H3K27me3: trimethylation of lysine 27 on histone H3
HCP5: HLA class I histocompatibility antigen protein P5
HOTAIR: HOX transcript antisense RNA
ITGB2-AS1: ITGB2 antisense RNA 1
KCNQ1OT1: KCNQ1 opposite strand/antisense transcript 1
LINC-PINT: long intergenic nonprotein-coding RNA, P53-induced transcript
LincRNA: long intergenic noncoding RNA
Lnc-CHOP: C/EBP homologous protein, long noncoding RNA
LncRNA: long noncoding RNA
MALAT1: metastasis-associated lung adenocarcinoma transcript 1
MDSCs: myeloid-derived suppressor cells
MEG3: maternally expressed gene 3
MEKi: MEK inhibitors
MHENCR: melanoma highly expressed competing endogenous lncRNA for miR-425 and miR-489
MIAT: myocardial infarction-associated transcript
MIRAT: MAPK inhibitor resistance-associated transcript
MiRNA: micro-RNA
NcRNA: noncoding RNA
OIS: oncogene-induced senescence
Olfr29-ps1: olfactory receptor 29, pseudogene 1
Orilnc1: oncogenic RAS-induced lncRNA 1
OVAAL: ovarian adenocarcinoma amplified long noncoding RNA
PcG: polycomb group
PEG10: paternally expressed gene 10
RBP: RNA-binding protein
RMEL3: restricted to melanocyte 3
RPAD: RNase R treatment, polyadenylation, and poly(A)+ RNA depletion
SAMMSON: survival-associated mitochondrial melanoma-specific oncogenic noncoding RNA
SiRNA: small interfering RNA
SPRY4-IT1: Sprouty4-intronic transcript 1
SRA: steroid receptor RNA activator
TSS: transcription start site
TUG1: Taurine upregulated 1
UCA1: urothelial cancer associated 1
ZEB1-AS1: zinc finger E-box binding homeobox 1 antisense RNA 1

## 1 Introduction

Cutaneous melanoma (CM) is a malignant neoplasm that arises from melanocytes, representing the leading cause of skin cancer‐related deaths worldwide, and its incidence is constantly growing in industrialized countries [1]. Although surgery remains the definitive treatment for early‐stage CM [2], it is rarely curative for advanced CM; moreover, metastatic CM is characterized by a substantial unresponsiveness to conventional therapies, including chemotherapy and radiotherapy [3]. A recent analysis of whole genome alterations in 183 CM samples indicated *BRAF* and *NRAS* as the most frequently mutated genes in CM [4]. In particular, approximately 50% of patients with CM harbor activating *BRAF^V600^* mutations, and in 90% of those mutations, a single nucleotide alteration (nucleotide 1799T>A) results in single amino acid substitution of valine by glutamic acid (*BRAF^V600E^*) [5]. In these patients, the constitutive activation of MAPK signaling caused by *BRAF^V600^* appears as a major driver of CM tumorigenic potential and survival [6]. Accordingly, *BRAF^V600^* mutation is an important factor to guide CM treatment, and BRAF and MEK inhibitors (BRAFi/MEKi) represent the best therapeutic strategy for *BRAF*‐mutated CM patients so far. In fact, the first‐line therapy with BRAFi, alone or in combination with MEKi, has shown remarkable response rates and a significantly improved progression‐free and overall survival in the advanced disease [7]. Despite these findings, about 15% of CM patients do not achieve tumor regression, due to primary resistance to BRAFi/MEKi, and progress more rapidly [8]. In addition, about 50% of CM patients, who initially respond to targeted therapy, ultimately develop an acquired resistance within 7 months from the start of the treatment [9].

The landscape of therapeutic strategies for CM has been revolutionized with the development of a new class of immune modulators, including checkpoint inhibitors targeting CTLA‐4 and PD‐1, which have demonstrated to provide durable responses in the metastatic disease regardless of mutation status [10]. However, primary resistance to immune checkpoint blockade occurs in approximately 40–65% of CM patients treated with PD‐1‐targeting therapy and in about 70% of those treated with anti‐CTLA‐4 therapy [11]. Furthermore, late relapses were also reported, suggesting the emergence of acquired resistance; indeed, 43% of CM responders to anti‐PD‐1 immunotherapy develop acquired resistance by 3 years [12]. Therefore, to advance in this field, novel targets and therapeutic approaches for more effective and long‐lasting treatments for CM patients must be explored. Although noncoding RNAs (ncRNAs) were for years considered as an irrelevant part of the genome, they have recently emerged as important modulators of several cancers [13, 14, 15], including CM [16, 17, 18, 19, 20, 21, 22, 23, 24, 25], and found to act as mediators of drug resistance mechanisms [26].

Based on these considerations, this review will provide novel insights on the function of selected circular RNAs (circRNAs) and long noncoding RNAs (lncRNAs) in *BRAF^V600^*‐mutant CM and in CM immunogenicity (Table S1). In addition, we present software algorithms currently available for the prediction and validation of the functional interactions of circRNAs and lncRNAs.

## 2 CircRNAs and lncRNAs

**Fig. 1:**
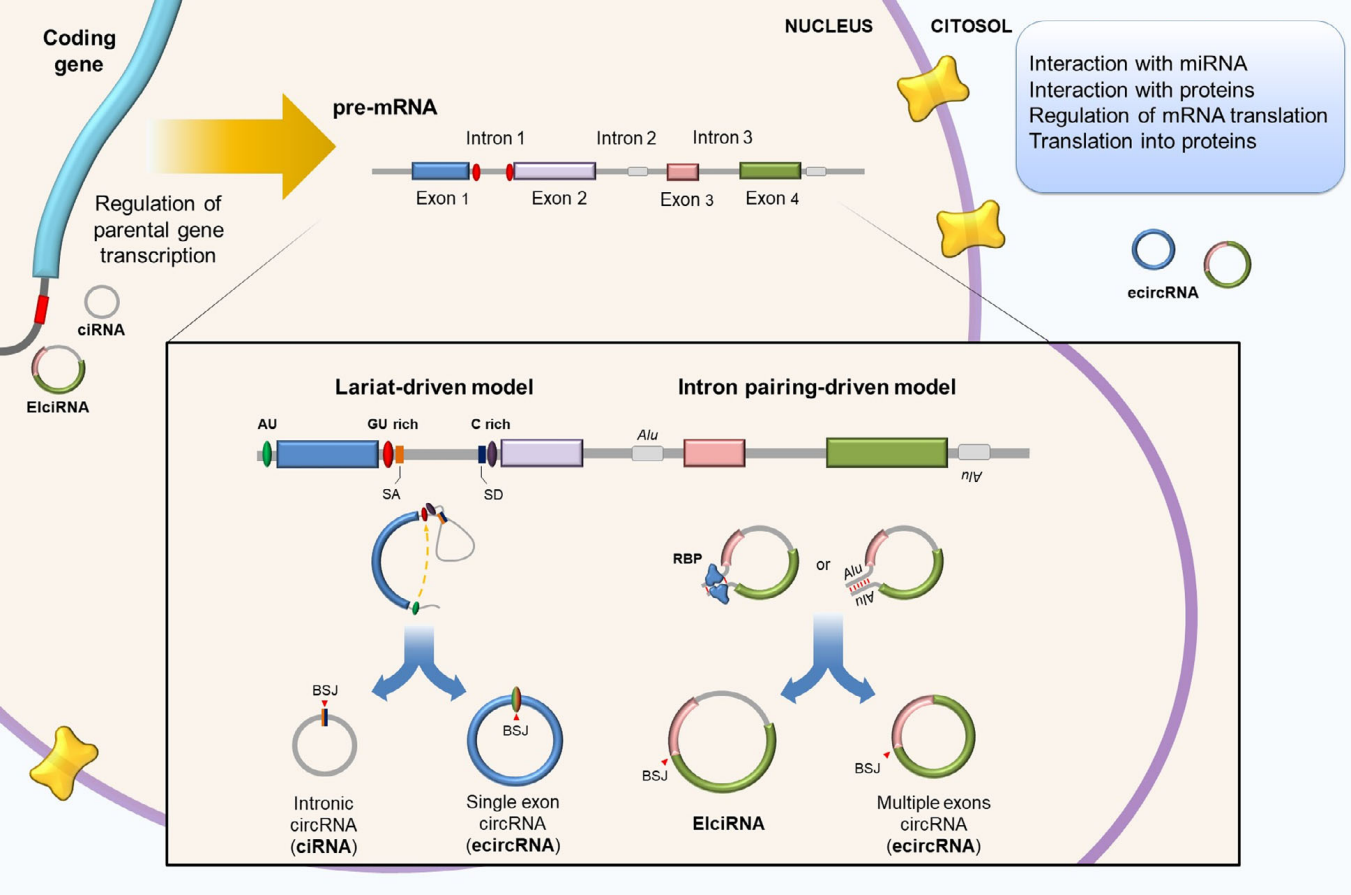
Biogenesis of circRNAs. During mRNA maturation, competition between linear splicing and backsplicing can lead to the formation of intron lariats, which can be further processed into circRNAs. Alternatively, the presence across flanking introns or within them of repeated sequences (i.e., Alu repeats with opposite directions) can produce intron‐driven circularization of RNA. In both lariat‐pairing‐driven circularization and intron‐pairing‐driven circularization, introns can be removed to originate an exonic circRNA (ecircRNA), or retained to form an intron‐containing circRNA (ciRNA or EIciRNA). CiRNA biogenesis relies on a consensus motif of a 7 nucleotide GU‐rich element near the 5′ spliced site and an 11 nucleotide C‐rich element adjacent to the branchpoint site. RNA‐binding proteins (RBPs) may actively participate in this process. EcircRNAs (exonic circRNAs) are mainly distributed in the cytoplasm, whereas ciRNAs (circular intronic RNAs) and EIciRNAs (exon‐ and intron‐containing circular RNAs) are primarily located in the nucleus.

CircRNAs are circular loop structures with covalently linked ends that are mainly generated by pre‐mRNA backsplicing, which connects a downstream 5′ splice donor site to an upstream 3′ splice acceptor site [27]. Due to their circular structure, circRNAs are more resistant to exonucleases that typically degrade linear RNA and much more stable in biological fluids [28]. CircRNAs are predominantly localized in the cytoplasm, whereas a limited number of circRNAs reside in the nucleus [29]. Exonic circRNAs (ecircRNAs) represent more than 80% of total circRNAs, are mainly cytoplasmic, and in some cases are expressed higher than their corresponding linear mRNAs [30]. CircRNAs can also arise from intron lariats that escape degradation after canonical splicing (ciRNAs) or from introns that have been retained between circularized exons (EIciRNAs), and both are primarily located in the nucleus, where they regulate the expression of their parental genes [31]. So far, two models of ecircRNA and EIciRNA formation have been proposed: the lariat‐driven circularization and the intron‐pairing‐driven circularization which differ for the order in which canonical and backsplicing occur [30] (Fig. 1).

LncRNAs are expressed at lower levels in comparison with mRNAs and display more tissue‐specific expression patterns [32]. Additionally, lncRNAs can be distributed either in the nucleus or the cytoplasm, or in both compartments simultaneously [summarized in [33]], and may or may not be subject to polyadenylation or alternative splicing [34]. Although few lncRNAs have been characterized in detail, it is clear that lncRNAs regulate various biological processes [35] in a number of different ways [summarized in [36]]. Based upon their genomic location, lncRNAs can be classified into five categories: (a) sense or (b) antisense, when the lncRNA overlaps the neighboring protein‐coding gene on the same, or opposite, strand, respectively; (c) bidirectional, when the lncRNA transcription start site (TSS) is located within 1 kb, but on the opposing strand, of the TSS of the nearest protein‐coding gene; (d) intronic, when lncRNA derives from intronic regions of protein‐coding genes; and (e) intergenic, or long intergenic noncoding RNAs (lincRNAs), when lncRNA is located within the genomic interval between two genes [37].

**Fig. 2:**
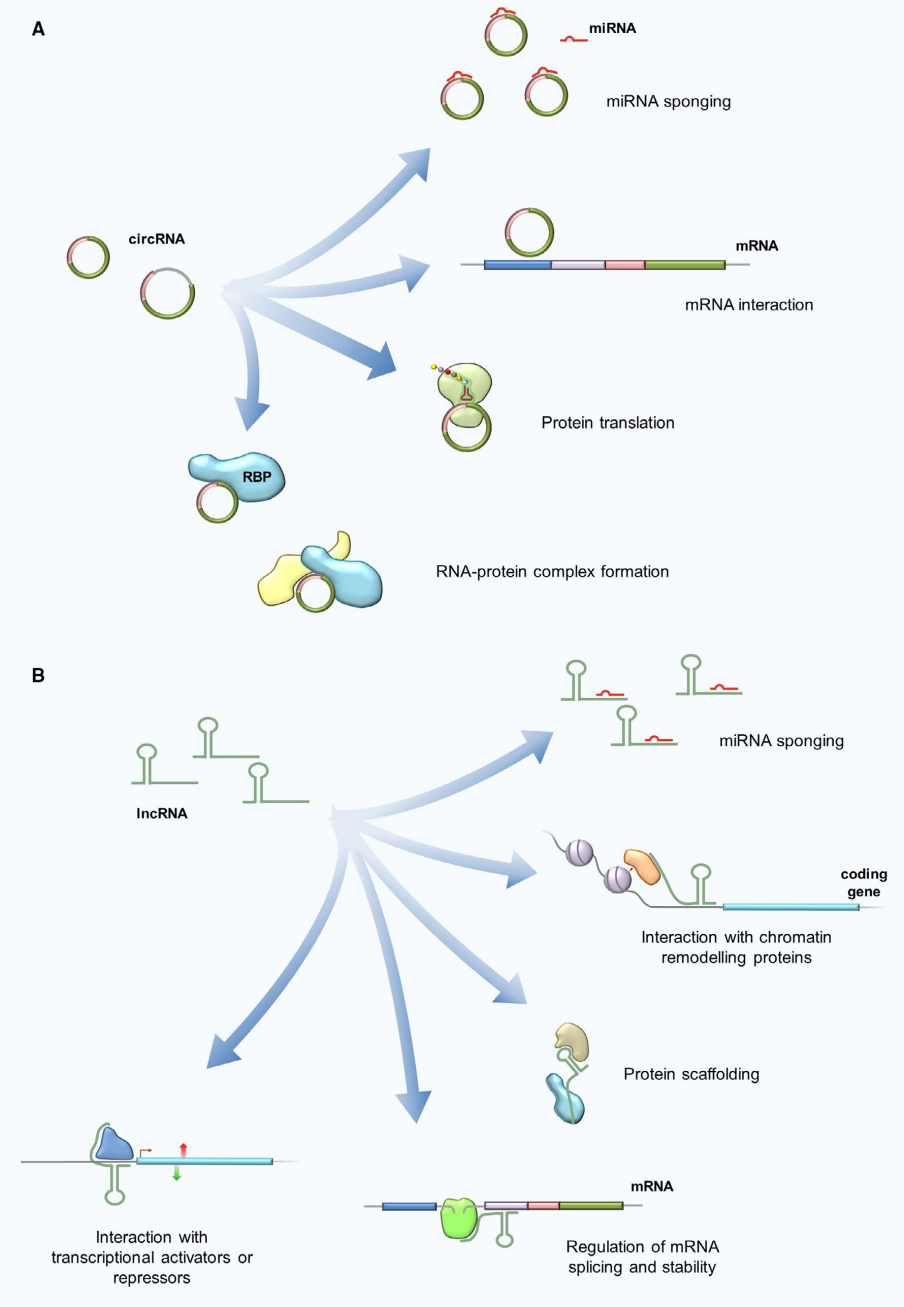
CircRNA (A) and lncRNA (B) functions. CircRNAs can modulate gene expression at different levels: by competitive miRNA sponging and sequestration, thus indirectly enabling the transcription of downstream genes, or by direct interaction with target mRNAs. In rare cases, circRNAs can be translated into proteins. Lastly, circRNAs can interact with RNA‐binding proteins (RBPs) to regulate multiple signaling pathways. LncRNAs are involved in transcriptional and post‐transcriptional regulation of gene expression. In particular, lncRNAs have been implied in different regulatory mechanisms: by competitively binding to miRNAs, by binding and redirecting chromatin remodeling proteins or transcription factors to alternatively modulate transcription of target genes, and by regulating mRNA splicing and degradation. In addition, lncRNAs can serve as scaffold for the formation of multiprotein complexes.

Besides acting as competitive endogenous RNAs (ceRNAs), both circRNAs and lncRNAs can also act through different mechanisms, as shown in Fig. 2. Interestingly, circRNAs or lncRNAs might also originate from chromosomal DNA translocations. However, the expression patterns and functions of these ncRNAs in solid tumors are still unclear [summarized in [38]].

## 3 LncRNAs as regulators of the MAPK‐signaling cascades

**Fig. 3:**
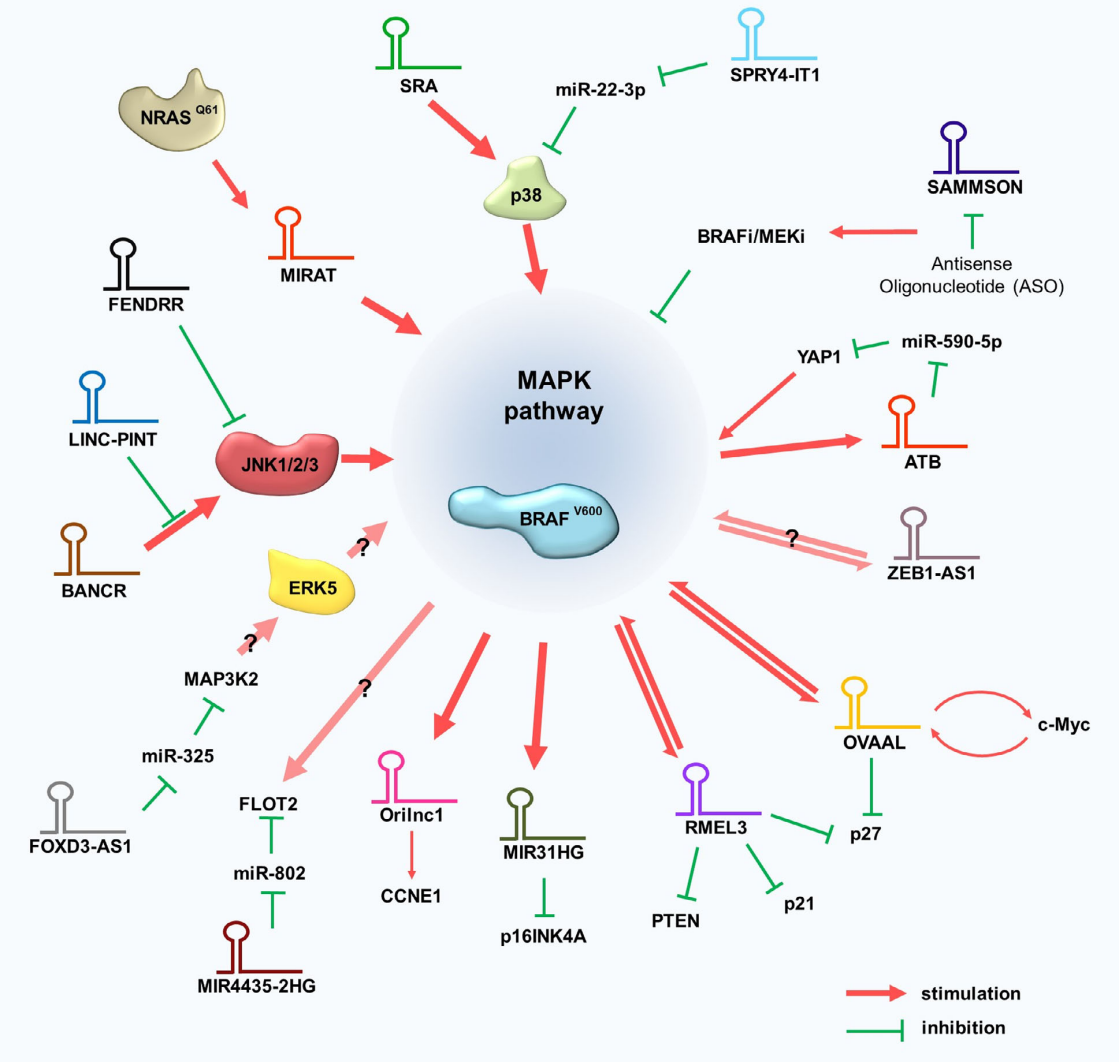
LncRNAs associated with the MAPK pathways in CM. Red arrows and green blocking bars indicate a positive or negative regulation, respectively.

MAPK pathways are cascades of four kinases that regulate a range of biological processes [summarized in [39, 40]]. So far, there are a number of studies aimed at elucidating lncRNA‐MAPK‐signaling interaction networks in CM harboring *BRAF* or *RAS* mutations, whereas no information on circRNAs is available (Table 1, Fig. 3).

**Table 1:** CircRNAs and lncRNAs that are aberrantly expressed in BRAF/RAS‐mutant CM.

Functional pathway	NcRNA ID	Expression change	Cell lines	BRAF/RAS mutational status	Target gene(s)	Notes	References
MAPK/ERK pathway	ATB	Up	A375 A2058	BRAF^V600E^	MiR‐590‐5p/YAP‐1		[119]
	BANCR	Up	A375 1205Lu SK‐MEL‐5	BRAF^V600E^	ERK1/2 and JNK pathway components	BANCR expression is downregulated by LINC‐PINT	[43, 44]
	MIR31HG	Up	Human diploid fibroblasts expressing a constitutively activated form of the mouse BRAF^V600E^ fused to the estrogen receptor	BRAF^V600E^	p16INK4A		[48]
	MIR4435‐2HG	Up	A375 A2058	BRAF^V600E^	MiR‐802/FLOT2 (MAPK/ERK?)		[57]
	MIRAT	Up	DO4 MM415	NRAS^Q61L^	MAPK pathway/IQGAP1		[61]
	Orilnc1	Up	A2058 LOX‐IMVI UACC‐257 WM9 WM983B 1205Lu 451Lu	BRAF^V600E^	Cyclin E1		[51]
			SK‐MEL‐2 WM3936	NRAS^Q61L^			
	OVAAL	Up	ME4405	NRAS^Q61L^	p27		[47]
	RMEL3	Up	WM278 WM1617	BRAF^V600E^	MAPK pathway components		[49]
	ZEB1‐AS1	Up	TGCA data	BRAF^V600E^ NRAS^Q61L^	MAPK/ERK?		[52]
p38/JNK pathway	BANCR	Up	A375 1205Lu SK‐MEL‐5	BRAF^V600E^	ERK1/2 and JNK pathway components	BANCR expression is downregulated by LINC‐PINT	[43, 44]
	FENDRR	Down	A375 SK‐Mel‐28	BRAF^V600E^	MMP2, MMP9, JNK pathway component		[67]
			SK‐MEL‐110	KRAS^E63K^			
	SPRY4‐IT1	Up	A375 WM1552C	BRAF^V600E^	MiR‐22‐3p/p38MAPK/MAPKAPK/Hsp27		[73]
	SRA	Up	A375 SK‐MEL‐1	BRAF^V600E^	p38		[74]
ERK5 pathway	FOXD3‐AS1	Up	A375 SK‐Mel‐1	BRAF^V600E^	MiR‐325/MAP3K2 (ERK5?)		[79]
PI3K/AKT pathway	H19	Up	C32 SK‐MEL‐28	BRAF^V600E^	PI3K/AKT and NF‐kB pathway components		[200]
	LINC00961	Down	A375 SK‐MEL‐28	BRAF^V600E^	MiR‑367/PTEN		[89]
	MHENCR	Up	A375	BRAF^V600E^	MiR‐425/489/PI3K‐Akt pathway		[85]
			SK‐MEL‐2	NRAS^Q61L^			
	MIAT	Up	A375 A2058 M21 SK‐MEL‐28	BRAF^V600E^	PI3K‐Akt pathway components		[158]
	PEG10	Up	A375	BRAF^V600E^	MiR‐33a/PI3K‐Akt and mTOR pathways		[83]
	RMEL3	Up	WM278 WM1617	BRAF^V600E^	PI3K/Akt pathway components		[49]
GAS6/AXL pathway	GAS6‐AS2	Up	A375 SK‐MEL‐5	BRAF^V600E^	GAS6, AXL		[94]
			SK‐MEL‐2	NRAS^Q61L^			
MITF pathway	DIRC3	Down	SK‐MEL‐28 A375 501mel	BRAF^V600E^	IGFBP5		[100]
PRC2 complex	ANRIL	Up	A375	BRAF^V600E^	CDKN2A/B		[130]
	CDR1as	Up	Cancer Cell Line Encyclopedia	BRAF^V600E^	IGF2 mRNA‐binding protein 3	CD1R arises from the PRC2‐mediated epigenetic silencing of the lncRNA LINC00632	[128]
	CircANRIL	Up	BJ	BRAF^V600E^	PRC proteins		[129]
	GAS5	Down	A375	BRAF^V600E^	EZH2		[135]
			SK‐MEL‐110	KRAS^E63K^			
	PVT1	Up	A375 SK‐MEL‐5	BRAF^V600E^	MiR‐200c/EZH2		[137]
EMT/invasion/metastasis	BANCR	Up	A375 A875 M14	BRAF^V600E^	MiR‐204/Notch2		[201]
	CASC2	Down	A375	BRAF^V600E^	MiR‐18a‐5p/RUNX1		[202]
			A375 M14	BRAF^V600E^	MiR‐181a/PLXNC1		[203]
	Circ_0016418	Up	SK‐MEL‐1 SK‐MEL‐5	BRAF^V600E^	MiR‐625/YY1		[115]
	Circ_0084043	Up	A375 A875	BRAF^V600E^	MiR‐153‐3p/Snail		[119]
			A375 SK‐MEL‐28	BRAF^V600E^	Wnt/β‐catenin pathway through miR‐429/TRIB2 axis		[120]
	CRNDE	Up	A375 M14	BRAF^V600E^	MiR‐205/CCL18		[204]
	GAS5	Down	A375 M21 SK‐Mel‐28	BRAF^V600E^	MMP2, MMP9		[205]
			SK‐Mel‐110	KRAS^E63K^			
	HOTAIR	Up	A375	BRAF^V600E^	MMP2, MMP9		[102]
			A375 A875 SK‐MEL‐1 SK‐MEL‐5 SK‐MEL‐28	BRAF^V600E^	MiR‐152‐3p/c‐MET		[104]
	KCNQ1OT1	Up	A375 A875 MuM‐2C	BRAF^V600E^	MiR‐153/c‐MET		[206]
	LINC00173	Up	A375 A2058 HT144 SK‐MEL‐1	BRAF^V600E^	MiR‐493/IRS4		[207]
	LINC00518	Up	A375 A2058 SK‐MEL‐28	BRAF^V600E^	MiR‐204‐5p/AP1S2		[112]
	LINC00963	Up	A375 A2058	BRAF^V600E^	MiR‐608/NACC1		[208]
	MALAT1	Up	A375 SK‐MEL‐5	BRAF^V600E^	MiR‐22/MMP14/Snail		[209]
			SK‐MEL‐2	NRAS^Q61L^			
	MIAT	Up	A375 SK‐MEL‐28	BRAF^V600E^	MiR‐150		[210]
	NEAT1	Up	A375 A2058 SK‐MEL‐28	BRAF^V600E^	MiR‐495‐3p/E2F3		[211]
			A375 A875 A2058 M14 451LU	BRAF^V600E^	MiR‐23a‐5p/KLF3		[144]
	MEG3	Down	A375 A875	BRAF^V600E^	MiR‐499‐5p/CYLD		[126]
					MiR‐21/E‐cadherin		[127]
	SSATX	Up	A375 A875	BRAF^V600E^	Wnt/β‐catenin pathway	Alternative splicing variant of the SAT1 gene, it might function as a lncRNA prior to its degradation	[212]
	SLNCR1	Up	A375	BRAF^V600E^	MMP9		[213]
	TUG1	Up	A375 SK‐MEL‐5 WM35	BRAF^V600E^	MiR‐129‐5p/AEG‐1		[123]
			SK‐MEL‐2	NRAS^Q61L^			
			A375	BRAF^V600E^	MiR‑29c‑3p/RGS1		[214]
			SK‐MEL‐2	NRAS^Q61L^			
	UCA1	Up	A375	BRAF^V600E^	MiR‐507/FOXM1		[215]
			SK‐MEL‐2	NRAS^Q61L^	MiR‐185‐5p/Wnt/β‐catenin pathway		[122]
			A375 A2058 HS294T WM266‐4	BRAF^V600E^			
Metabolism	CircMYC	Up	Mel‐CV	BRAF^V600E^	MiR‐1236/LDHA	c‐MYC‐SRSF1 axis regulates the production of circMYC	[141]
	Circ_ITCH	Down	A375 M21	BRAF^V600E^	GLUT1	Circ_ITCH is generated from several exons of ITCH	[140]
	Circ_0016418	Up	A375 A875	BRAF^V600E^	MiR‐605‐5p/GLS		[145]
	Circ_0025039	Up	A375 A2058 SK‐MEL‐1	BRAF^V600E^	MiR‐198/CDK4	Circ_0025039 originates from the NM_202002 fragment of chromosome 12, which is homologous to the protein‐coding gene FOXM1	[142]
	Circ_0084043	Up	A375 A378	BRAF^V600E^	MiR‐31/KLF3 axis		[143]
	H19	Up	A375 SK‐MEL‐1 SK‐MEL‐5	BRAF^V600E^	MiR‐106a‐5p/E2F3		[216]
	OIP5‐AS1	Up	A375	BRAF^V600E^	MiR‐217/GLS		[146]

### 3.1 LncRNAs related to MAPK/ERK signaling pathway

As stated above, the MAPK/ERK cascade plays a key role in *BRAF^V600^*‐mutant CM development, making it the most prominent and clinically utilized therapeutic target [summarized in [41]]. In this context, a number of lncRNAs were shown to actively interact with BRAF^V600^ and/or MAPK/ERK pathway in CM, including the oncogenic *BRAF*‐activated nonprotein‐coding RNA (BANCR). BANCR was originally correlated with BRAF^V600^ activation since it was found to be overexpressed in *BRAF^V600^*‐mutant CM in comparison with normal melanocytes. Although BANCR was initially described as a regulator of CM migration [42], subsequent studies demonstrated that BANCR regulated CM progression through activating the ERK1/2 and JNK/MAPK pathways both *in vitro* and *in vivo* [43]. In *BRAF*‐mutant A375 cells, BANCR expression appeared to depend on the long intergenic nonprotein‐coding RNA p53‐induced transcript (LINC‐PINT) [44], which is known to function as a tumor suppressor [45] and to interact with MAPK [46]. Despite these findings, the question of whether LINC‐PINT might regulate the BANCR/MAPK axis to inhibit *BRAF*‐mutant CM progression deserves further study.

In their study, Sang *et al*. reported a significant upregulation of the lncRNA ovarian adenocarcinoma amplified long noncoding RNA (OVAAL) in *BRAF*‐mutant CM compared with wild‐type CM in a TCGA dataset. Detailed mechanistic insights revealed that OVAAL was bound to STK3, enhanced the structural association of STK3 with Raf‐1, and activated the MAPK/ERK signaling pathway which, in turn, promoted c‐Myc‐driven proliferation. Following treatment with the MEKi UO126, OVAAL failed to influence c‐Myc levels, thus confirming that the OVAAL‐mediated upregulation of c‐Myc was depended on the MAPK pathway. In addition, silencing of c‐Myc reduced, whereas overexpression of c‐Myc increased, OVAAL expression levels. These results clearly suggested a positive feedback loop between c‐Myc, OVAAL, and MAPK/ERK signaling pathway in controlling tumor growth [47]. The same group reported that OVAAL competed with p27 mRNA for binding to PTBP1, thus impairing p27 mRNA translation and allowing CM cells to escape from cellular senescence [47]. However, since these studies have been performed in *BRAF^WT^*/*NRAS^Q61R^* CM cells, it would be of interest to address in more detail the specific role of OVAAL in *BRAF*‐mutant CM.

Alike to OVAAL, the lncRNA MIR31HG was implicated in CM senescence. Intriguingly, both activity and subcellular localization of MIR31HG were strictly dependent on *BRAF^V600E^* [48]. Under normal conditions, MIR31HG was predominantly located in the nucleus of CM cells, where it recruited polycomb group (PcG) proteins to the *INK4* locus to repress p16INK4A expression. Interestingly, MIR31HG knockdown reduced PcG chromatin occupancy and induced p16INK4A‐dependent senescence, which was reverted by MIR31HG overexpression. RNA‐seq analysis of CM samples holding normal diploid *INK4A* loci revealed a negative correlation between MIR31HG and p16INK4A expression, indicating that MIR31HG‐mediated repression of p16INK4A might drive CM progression. The same authors also observed that following *BRAF^V600E^* activation, MIR31HG translocated to the cytoplasm, whereas CM cells acquired an oncogene‐induced senescence (OIS) phenotype along with an increased expression of p16INK4A protein. Consistent with this, MIR31HG depletion reduced *BRAF^V600E^* CM cell growth and promoted OIS. However, although p16INK4A levels decreased upon MIR31HG overexpression, OIS was not reverted, thus highlighting the complexity of molecular mechanisms involved in *BRAF^V600E^*‐induced senescence [48].

Compared with wild‐type CM, *BRAF^V600^*‐mutant CM exhibited a significant upregulation of the oncogenic restricted to melanocyte 3 (RMEL3) [49]. Enforced RMEL3 expression enhanced *BRAF^V600^*‐mutant CM cell proliferation and clonogenic ability both *in vitro* and *in vivo* [50], whereas RMEL3 abrogation decreased cell survival and proliferation along with an increase in PTEN and cell cycle inhibitors p21 and p27 protein levels [49]. Aberrant expression levels of MAPK/ERK and PI3K/AKT pathway effectors were also observed upon RMEL3 silencing [49], thus indicating that MAPK/ERK activation and RMEL3 expression might be coordinately regulated through a positive feedback loop. Similarly to RMEL3, the oncogenic *RAS*‐induced lncRNA 1 (Orilnc1) was found increased in *BRAF^V600^*‐mutant CM in respect to wild‐type CM. In line with the observation that Orilnc1 was induced by RAS‐RAF‐MEK‐ERK pathway activation, Orilnc1 acted as a mediator of RAS signaling and promoted an oncogenic CM phenotypes by regulating cyclin E1 in *BRAF*‐mutant CM cells [51]. In addition to RMEL3 and Orilnc1, CM samples carrying *BRAF* or *NRAS* mutations overexpressed the lncRNA *ZEB1* antisense RNA 1 (ZEB1‐AS1) [52]. However, the correlation between ZEB1‐AS1 deregulation and MAPK activation in *BRAF*‐mutant CM has not been assessed so far.

The lncRNA activated by TGF‐beta (ATB) could enhance the expression of YAP‐1 by sponging miR‐590‐5p to promote proliferation, migration, and invasion of *BRAF*‐mutant CM cells. Of note, YAP‐1 activation induced the ERK/MAPK‐signaling pathway in gallbladder [53] and papillary thyroid cancers [54] which commonly harbor *BRAF* mutations [55, 56]. Hence, it would be interesting to study ATB regulation and to investigate whether the ATB/YAP‐1 axis triggers the ERK/MAPK pathway in *BRAF*‐mutant CM.

Recently, a positive correlation between MIR4435‐2HG and flotillin 2 (FLOT2) expression was identified in A375 and A2058 cells, where MIR4435‐2HG sponged miR‐802 to upregulate FLOT2 [57]. Small interfering RNA (siRNA) targeting FLOT2 restrained A375 cell proliferation, migration, and invasion, whereas MIR4435‐2HG upregulation or miR‐802 silencing abrogated the inhibitory effects of FLOT2 knockdown. FLOT2 is highly expressed in CM and was related to lymph node CM metastasis [58]. Of interest, MAPK/ERK pathway was predicted to play a key role in the signaling cascade caused by FLOT2 overexpression in CM [59]. Hence, further investigation is needed to determine whether MIR4435‐2HG/miR‐802/FLOT2 axis might affect *BRAF*‐mutant CM progression through modulating MAPK/ERK signaling.

The above reported data clearly indicate that altered lncRNA expression contributes to the abnormal MAPK signaling in *BRAF^V600^*‐mutant CM. In addition, considering that MAPK pathway activation represents a frequent mechanism of resistance for small molecules directed against BRAF^V600^, it is reasonable to believe that aberrant lncRNA expression might also influence CM resistance to BRAFi. Actually, little is known about the role of lncRNAs in the establishment of CM resistance to BRAFi [60], and only few studies have explored the impact of lncRNA silencing in restoring sensitivity to target therapies in CM. For instance, an induced expression of the lncRNA‐MAPK inhibitor resistance‐associated transcript (MIRAT) was found in *NRAS*‐mutant CM cells with acquired resistance to BRAFi/MEKi [61]. Gain‐ and loss‐of‐function assays, as well as RNA–protein interaction assays, indicated that MIRAT modulated the MAPK‐signaling pathway by binding to the scaffold protein IQGAP1 [61], which could promote RAS‐MAPK‐driven cancer invasion [62]. Interestingly, MIRAT depletion did not significantly affect cell viability in resistant *NRAS*‐mutant CM cells, thus suggesting that, despite its role in regulating MAPK activation, MIRAT silencing was not sufficient to revert resistance to targeted therapy [61]. However, since no additional studies have been performed correlating MIRAT with BRAFi/MEKi resistance in *BRAF*‐mutant CM, this lncRNA should be further investigated. Differently, the silencing of the lncRNA survival‐associated mitochondrial melanoma‐specific oncogenic noncoding RNA (SAMMSON) drastically impaired CM cell viability irrespective of their *BRAF*, *NRAS*, or *p53* mutational status and improved sensitivity toward targeted therapy in patient‐derived xenograft models of *BRAF^V600^*‐mutant CM [63]. Mechanistically, SAMMSON interacted with p32 to maintain its mitochondrial localization and to enhance its function. Concordantly, SAMMSON targeting using antisense oligonucleotides (ASOs) decreased mitochondrial ribosome biogenesis, oxidative phosphorylation, and respiratory chain complex activity. Therefore, the synergistic killing of *BRAF^V600^*‐mutant CM cells observed upon co‐targeting of SAMMSON and MAPK pathway components was likely to arise because BRAFi elevated oxidative phosphorylation [64], whereas SAMMSON silencing led to mitochondrial dysfunction.

### 3.2 LncRNAs related to JNK and p38 MAPK‐signaling pathways

Besides MAPK/ERK cascade, stress‐activated MAPK pathways, such as JNK and p38, play important modulatory roles that can influence the response of CM cells to targeted therapy [65, 66]. For instance, as reported above, BANCR was demonstrated to activate JNK along with MAPK/ERK signaling pathway [43]. Another lncRNA, namely *FOXF1* adjacent noncoding developmental regulatory RNA (FENDRR), mediated proliferation, migration, and invasion of *BRAF*‐ and *KRAS*‐mutated CM cells through the JNK pathway. Contrary to BANCR, FENDRR was downregulated in CM with the lowest expression in CM with metastasis [67]. *In vitro* and *in vivo* functional analyses revealed that FENDRR not only antagonized the JNK pathway, but also inhibited matrix metallopeptidases expression. So far, three different JNK isoforms have been identified, namely JNK1, JNK2, and JNK3. Interestingly, a paper of Du *et al*. [68] reported that JNK2 expression was significantly higher than JNK1 in CM and was specifically required for cell proliferation, invasiveness, and adaptive BRAFi resistance. However, the studies of Li *et al*. and Chen *et al*. did not indicate which JNK isoform interacted with BANCR and FENDRR, respectively.

Other studies reported that p38/MAPK might mediate cell survival [69] or cell death in *BRAF*‐mutant CM [70] depending on the cell context and the type of stimulus. For instance, p38/MAPK signaling was reported to be involved in biological processes associated with CM progression and mediated by the lncRNAs *SPRY4* intronic transcript 1 (SPRY4‐IT1), which was initially identified to be upregulated in *BRAF*‐mutant WM1552C and A375 cells in comparison with melanocytes. SiRNA‐mediated SPRY4‐IT1 knockout was shown to inhibit CM cell proliferation, motility, and invasion, while increasing apoptosis [71]. SPRY4‐IT1 is transcribed from the second intron of the *SPRY4* gene, a regulator of the MAPK cascade [72], indicating that SPRY4‐IT1 may also affect the MAPK‐signaling pathway. To investigate this further, A375 cells were transfected with short hairpin RNA targeting SPRY4‐IT1. Results demonstrated that SPRY4‐IT1 depletion reduced the phosphorylation levels of p38, MAPKAPK, and Hsp27. In addition, SPRY4‐IT1 knockdown enhanced miR‐22‐3p levels and inhibited CM proliferation and metastasis. Hence, Li *et al*. [73] proposed that SPRY4‐IT1 could act as ceRNA via sponging miR‐22‐3p to activate the p38 MAPK‐signaling pathway in CM.

Alike to SPRY4‐IT1, the lncRNA steroid receptor RNA activator (SRA) was upregulated in A375 and SK‐MEL‐1, both of which are *BRAF*‐mutant CM cell lines. Functional assays showed that SRA mediated cell proliferation and regulated cell invasion in the A375 cell line and in B16 murine CM cells. Of interest, a shift from p38 to MEK1/2 and BRAF phosphorylation emerged in B16 cells when SRA was inhibited with siRNAs [74]. However, since B16 cells do not harbor a *BRAF* mutation, future studies would be necessary to further explore whether SRA influences MAPK signals in *BRAF*‐mutant CM.

### 3.3 LncRNAs related to ERK5 signaling pathway

*ERK5* was recently shown to be activated in *BRAF*‐mutant CM and to be involved in BRAFi/MEKi resistance [75, 76, 77, 78]. Of interest, the lncRNA *FOXD3* antisense RNA 1 (FOXD3‐AS1) sponged miR‐325 to positively regulate MAP3K2, an upstream activator of ERK5, in A375 and SK‐MEL‐1 cells. In addition, MAP3K2 overexpression could rescue the effect induced by FOXD3‐AS1 silencing and improved proliferation, invasion, and migration of *BRAF*‐mutant CM [79]. At the moment, however, it remains to be clarified whether the FOXD3‐AS1/miR‐325/MAP3K2 axis also affects the ERK5 pathway and/or has a role in targeted therapy resistance.

## 4 Pleiotropic effects of circRNAs and lncRNAs in *BRAF*‐mutant CM

In addition to MAPK‐related lncRNAs, several other circRNAs and lncRNAs have demonstrated aberrant expression in CM. Since these studies were mainly conducted in *BRAF*‐ and *RAS*‐mutant CM cell lines, circRNA and lncRNA deregulation likely represents a mechanism for strengthening the already activated MAPK signaling. Consistent with this hypothesis, most of these ncRNAs were proven to target molecular pathways that cooperate with MAPK family members and/or are known to be involved in BRAFi/MEKi resistance of CM cells. More importantly, restoration of their expression could revert the malignant phenotype both *in vitro* and *in vivo*, thus confirming their pathogenic relevance (Table 1).

### 4.1 LncRNA modulation of PI3K/AKT signaling

The PI3K/AKT signaling pathway is one of the major regulators of cell survival and apoptotic cell death. PI3K/AKT and MAPK/ERK pathways strictly regulate each other; therefore, the inhibition of one of these two pathways can promote the activity of the other one [80]. PI3K/AKT aberrant activation is a common phenomenon in CM cells, where increased PI3K/AKT signaling, with or without concomitant MAPK activity, represents an alternative path to both innate and acquired BRAFi/MEKi resistance [81].

Microarray analysis in 18 melanocytic nevi with and four nevi without the *BRAF^V600E^* mutation revealed 92 upregulated genes in nevi with the *BRAF* mutation, including the lncRNA paternally expressed gene 10 (PEG10) [82], thus suggesting that gain of PEG10 expression might occur early during *BRAF*‐mutant CM development. Functional analyses demonstrated that PEG10 silencing reduced cyclin D1 and CDK4 expression, triggered apoptosis, and impaired A375 CM cell migration and invasion. More specifically, PEG10 knockdown obstructed PI3K/AKT pathway by enhancing the expression of miR‐33a [83] which functions as a tumor suppressor in CM [84]. Although these results enforced PEG10 involvement in the progression of *BRAF^V600^* CM, the deeper correlation between PEG10 and PI3K/AKT pathway remains to be further explored.

Chen *et al*. [85] identified melanoma highly expressed noncoding RNA (MHENCR) as a critical regulator of PI3K/AKT. Mechanistically, MHENCR associated with miR‐425 and miR‐489 which inhibit PI3K/AKT pathway via targeting IGF1 and spindlin 1, respectively. PI3K/AKT activation through IGF1 deregulation has been shown to result in CM metastasis [86] and resistance to BRAFi‐induced apoptosis [81]. In patients with BRAFi resistance, deregulation of the PI3K/AKT pathway may be mediated by several mechanisms, including the loss of function of the tumor suppressor *PTEN* [87]. NcRNAs have been shown to regulate PTEN, thus contributing to the aberrant activation of the PI3K/AKT pathway. Among them, miR‐367 was reported to directly regulate PTEN protein expression to promote CM development [88]. Recently, Mu *et al*. provided the first evidence that the lncRNA LINC00961 acted as a micro‐RNA (miRNA) sponge for miR‐367. By sponging miR‐367, LINC00961 restored PTEN expression and suppressed migration and invasion of the *BRAF*‐mutant A375 and SK‐MEL‐28 cells [89]. Hence, whether MHENCR and LINC00961 are involved in CM resistance to BRAFi/MEKi requires further study.

### 4.2 LncRNA regulation of Gas6/AXL signaling pathway

GAS6 is a ligand for several receptor tyrosine kinases, including AXL which is usually highly expressed in BRAFi/MEKi‐resistant CM [90, 91, 92]. Furthermore, recent genomic and transcriptomic data from metastatic CM patients indicated that AXL overexpression might cause resistance to anti‐PD‐1 therapy [93]. In a recent study, Wen *et al*. found that the antisense RNA 2 of *GAS6* (GAS6‐AS2) promoted the secretion of GAS6 in the CM cell supernatants and further increased the phosphorylation levels of AXL, AKT, and ERK in an autocrine manner. In addition, GAS6‐AS2 accelerated CM cell proliferation, and inhibited CM cell apoptosis both *in vitro* and *in vivo*. Notably, ectopic expression of GAS6‐AS2 activated the pro‐survival GAS6/AXL/AKT/ERK signals not only in *BRAF^V600^* CM cells but also in *NRAS*‐mutant CM, thus supporting the rationale for further investigation on the potential implications of GAS‐AS2 in BRAFi/MEKi resistance [94].

### 4.3 LncRNAs in MITF signaling pathway

MITF is a master regulator transcription factor with well‐documented roles not only in melanocytes, but also in CM progression. Three major subpopulations of cells with different MITF expression levels have been detected in CM, some with high MITF levels, which were more proliferative, some exhibiting low MITF levels along with higher invasive and tumor‐forming capacities, and others expressing markers of both signatures [95]. In BRAFi/MEKi‐resistant CM cells, low MITF expression could induce high levels of tyrosine kinase receptors, such as AXL and EGFR, thus contributing to prolonged therapy resistance [92, 96]. However, MITF overexpression could also drive resistance, indicating its complex role in CM resistance to targeted therapy [97]. SOX10 activates *MITF* transcription in a *cis*‐acting fashion in melanocytes and CM [98] and cooperates with MITF in activating further downstream targets [99]. Coe *et al*. identified 245 CM‐associated lncRNAs whose loci were cobound by MITF and SOX10, including disrupted in renal carcinoma 3 (DIRC3). DIRC3 was described as a nuclear regulatory lncRNA that activated the expression of the neighboring *IGFBP5* tumor suppressor gene. DIRC3 loss of function in three *BRAF*‐mutant CM cell lines led to increased anchorage‐independent growth and SOX10 occupancy at putative regulatory elements within the *DIRC3* locus [100]. Furthermore, DIRC3 depletion enhanced SOX10‐mediated repression of *IGFBP5* [100], which negatively regulated MAPK kinase signaling to inhibit *BRAF*‐mutant A375 cell proliferation and metastasis [100].

### 4.4 CircRNA and lncRNA involvement in epithelial‐to‐mesenchymal transition (EMT), invasion, and metastasis

It is widely recognized that oncogenic *BRAF* and *RAS* modulate the expression of cell adhesion‐associated proteins and induce an EMT switch that promotes metastasis and CM progression [101]. Consistently, a close correlation between an EMT‐like phenotype and ncRNAs deregulation was found in CM cells carrying *BRAF* or *RAS* mutations. As shown in Table 1, deregulated circRNAs and lncRNAs can impact CM epithelial plasticity by affecting different target genes, and their effects are mainly ascribed to their ability to act as ceRNAs.

*HOX* transcript antisense RNA (HOTAIR) has emerged as a critical factor for CM metastatic state since its expression was dramatically increased not only in metastatic respect to primary CM [102], but also in lymphocytes surrounding metastatic CM cells [103]. Luan *et al*. [104] suggested that HOTAIR might promote CM invasion and migration by competitively binding to miR‐152‐3p to upregulate the tyrosine kinase c‐MET, which is known to be involved in CM metastasis [105]. The activation of c‐MET by the lncRNA *KCNQ1* opposite strand/antisense transcript 1 (KCNQ1OT1) was also found to increase the metastatic growth of A375 cells. Importantly, besides promoting CM metastasis, c‐MET upregulation was recognized to contribute to BRAFi resistance [106], whereas both HOTAIR and KCNQ1OT1 were supposed to play a role in chemoresistance [107, 108, 109] and radioresistance [110, 111]. Despite these findings, however, no study has demonstrated their possible involvement in c‐MET‐induced BRAFi resistance so far.

LINC00518 promoted *in vitro* invasion and migration of *BRAF*‐mutant A375 and A2058 cells and *in vivo* pulmonary metastasis through decoying miR‐204‐5p to upregulate AP1S2 expression [112]. Interestingly, a previous study demonstrated that *BRAF^V600^* negatively regulated miR‐204 through the MAPK/ERK pathway, whereas treatment with BRAFi/MEKi induced its expression. Furthermore, miR‐204 overexpression potentiated anti‐migratory activity of BRAFi‐resistant CM cells by targeting mRNA [113].

Using microarray analysis, several aberrantly expressed circRNAs were identified in the *BRAF*‐mutant WM35 and WM451 cell lines compared with normal melanocytes. Functional tests revealed that, among these circRNAs, circ_0000082, circ_0008157, circ_0016418, circ_0023988, and circ_0030388 regulated proliferation and invasion of CM cells [114]. Further research indicated that circ_0016418 contributed to SK‐MEL‐1 and SK‐MEL‐5 cell proliferation and metastasis in skin melanoma by sponging miR‐625 to activate YY1 [115]. Of note, Du *et al*. [116] uncovered that YY1 suppression enhanced antitumor efficacy of BRAFi both *in vitro* and *in vivo*. Nevertheless, whether circ_0016418/miR‐625/YY1 axis takes part in regulating the response to BRAFi is still unknown.

In a study by Luan *et al*., circRNA_0084043 was reported to directly bind to miR‐153‐3p, a tumor suppressor capable of regulating EMT through targeting SNAIL [117, 118]. The use of siRNA targeting circRNA_0084043 and miR‐153‐3p mimics significantly repressed proliferation, migration, and invasion abilities of *BRAF*‐mutant A375 and A875 cells. Furthermore, circRNA_0084043 knockdown decreased both mRNA and protein levels of SNAIL, and this inhibition was attenuated by cotransfection of a miR‐153‐3p inhibitor. Therefore, circRNA_0084043 might play a pivotal role in *BRAF*‐mutant CM progression via sponging miR‐153‐3p to upregulate SNAIL [119]. In a subsequent study, Chen *et al*. further evaluated the effects of circ_0084043 knockdown through *in vivo* and *in vitro* experiments that confirmed its oncogenic role in CM. In particular, the authors unveiled that circ_0084043 positively controlled TRIB2 expression through sponging miR‐429. Notably, the downregulation of TRIB2 following circ_0084043 knockdown not only reduced proliferation, migration, and invasion of *BRAF*‐mutant A375 and SK‐MEL‐28 cells, but also inhibited β‐catenin, c‐Myc, and cyclin D1 expression. These results highlighted the ability of circ_0084043/miR‐429/TRIB2 axis to control the Wnt/β‐catenin signaling pathway [120], which is frequently activated in EMT and metastasis [101], and was recently found to correlate with overall immune suppression and to drive immunotherapy resistance in CM as well [121]. Hence, the potential effects of ncRNAs/Wnt/β‐catenin network on resistance to both targeted agents and immune checkpoint inhibitors should be considered for future studies. Similarly to circ_0084043, the lncRNA urothelial carcinoma associated 1 (UCA1) modulated the expression of β‐catenin and c‐Myc through a competitive ceRNA network, leading to EMT in *BRAF*‐mutant CM cells [122]. Taurine upregulated 1 (TUG1) sequestered miR‐129‐5p to upregulate AEG‐1, a downstream target of Ras and c‐Myc [123]. The use of shRNAs targeting TUG1 alleviated the invasive and migratory abilities of A375 cells and inhibited AEG‐1 protein expression. Furthermore, effects of TUG1 silencing were abrogated by AEG‐1 cotransfection, thus confirming that TUG1 functions were mediated by AEG‐1. Of interest, Zhang *et al*. [124] have previously reported that ectopic expression and/or silencing of AEG‐1 influenced the expression of several EMT regulators through the Wnt/β‐catenin pathway, suggesting that TUG1 might indirectly regulate EMT and Wnt signaling through the miR‐129‐5p/AEG‐1 axis.

So far, a limited number of lncRNAs with metastatic suppressor function has been reported in CM, including maternally expressed gene 3 (MEG3) [125, 126, 127]. MEG3 restoration could limit EMT‐like phenotype in *BRAF*‐mutant CM cells through regulating E‐cadherin expression by targeting miR‐21 [127] and miR‐499‐5p, which negatively regulated CYLD [126]. Importantly, high levels of plasma MEG3 were linked with longer survival in BRAFi‐treated CM patients, whereas CYLD downregulation might protect CM cells from BRAFi/MEKi‐induced apoptosis. Hence, the role of MEG3/miR‐499‐5p/CYLD in CM resistance to BRAFi would require further evaluation.

### 4.5 CircRNA and lncRNA interaction with epigenetic complexes

Some ncRNAs have shown to affect the chromatin landscape of CM cells by interacting with epigenetic enzymes, and, in turn, they can be themselves targets of these epigenetic mediators. For instance, the circRNA *cerebellar degeneration‐associated protein 1* antisense transcript (CDR1as) has been proven to directly arise from the PRC2‐mediated epigenetic silencing of the lncRNA LINC00632 [128], whose function in CM has yet to be defined. Downregulation of CDR1as positively correlated with CM progression since CDR1as reduction resulted in CM invasion and metastasis by enhancing IGF2BP3. Interestingly, 18/21 cell lines with low CDR1as levels (CDR1as^low^) harbored *BRAF* mutation, suggesting that CDR1as loss might be required for pro‐metastatic functions of IGF2BP3 in *BRAF*‐mutant CM. Furthermore, CDR1as^low^ was more sensitive to several MAPK pathway inhibitors, suggesting that CDR1as expression levels might be a useful marker to predict the response to targeted therapy [128].

Antisense noncoding RNA in the *INK4* locus (ANRIL) is a well‐established example of lncRNA that interacts with PRC2 to mediate epigenetic silencing of p15^INK4b^ and p16^INK4a^ genes [129]. ANRIL was highly expressed in *BRAF*‐mutant A375 and OM431 cell lines, and its silencing activated p15^INK4b^ and p16^INK4a^ expression, thus significantly reducing CM growth both *in vitro* and *in vivo* [130]. Recently, Sakar *et al*. described several circular isoforms of the ANRIL, called circANRIL, which were all expressed in the cytoplasm of CM cell lines, thus suggesting their involvement in post‐transcriptional regulatory mechanisms. Importantly, since the expression of the linear ANRIL was specifically enriched in the nucleus, these results also indicated divergent activities for linear and circular isoforms of ANRIL [131]. Consistent with this hypothesis, a study of Muniz *et al*. speculated that, in proliferative cells, ANRIL would prevent senescence by repressing *INK4* locus through PRC2 recruitment. On the contrary, during BRAF‐ and MEK‐induced senescence, circular ANRIL species would sequester PRC2 proteins in the cytoplasm to prevent them from being recruited to the *INK4* locus [129].

PRC2 contains different catalytic components, including the histone methyltransferase EZH2 that catalyzes the trimethylation of histone H3 lysine 27 (H3K27me3) [132]. EZH2 has been evidenced to have a crucial role in CM progression [133], especially in *BRAF*‐mutant CM where *BRAF^V600^* mutation and EZH2 gain often coexist [134]. Mechanistic investigations revealed that the silencing of the lncRNA GAS5 accelerated EZH2 expression to suppress the transcription of *CDKN1C* in A375 *BRAF*‐mutant CM cells [135]. On the other hand, when overexpressed, GAS5 inhibited EZH2, prevented H3K27me3, and upregulated CDKN1C expression, thus suppressing CM cells viability, and inducing apoptosis and oxidative stress [135]. Oxidative stress is a cellular characteristic of CM that has acquired BRAFi resistance and that likely renders them more sensitive to pro‐oxidative agents [136]. Hence, further studies are warranted to clarify whether the GAS5/EZH2 axis is implicated in the oxidative state of CM resistant to BRAFi.

By using *BRAF*‐mutant SK‐MEL‐5, Chen *et al*. [137] discovered that the oncogenic plasmacytoma variant translocation 1 (PVT1) directly bound to EZH2 in order to epigenetically inhibit the expression of miR‐200c, which has been described as a potential therapeutic target for overcoming BRAFi resistance [138]. In fact, loss of miR‐200c expression was found to promote a BRAFi‐resistant phenotype in CM cells and tissues with a mechanism that involved both MAPK and PI3K/AKT signaling pathways [138]. Therefore, PVT1 might be a key molecule in the development of BRAFi resistance in CM.

### 4.6 CircRNAs and lncRNAs as metabolism regulators

*BRAF* mutation dramatically affects CM metabolism, depending mainly on glycolytic metabolism [summarized in [139]]. In this context, ncRNAs were found to regulate glucose metabolism and lactate production in *BRAF*‐mutant CM cells. For example, the overexpression of a circRNA namely circ_ITCH restrained glucose uptake in *BRAF*‐mutant A375 and M21 cell lines, thereby preventing CM cell proliferation. Notably, circ_ITCH did not act as a miRNA sponge since it directly downregulated glucose transporter 1 expression [140]. On the other hand, circMYC was shown to promote Mel‐CV proliferation and to accelerate glycolysis by binding to miR‐1236, a negative regulator of lactate dehydrogenase A. CircMYC silencing significantly decreased lactate production, whereas its overexpression generated opposite effects [141]. Evidence from both *in vitro* and *in vivo* studies revealed that circ_0025039 also facilitated glucose metabolism in *BRAF*‐mutant CM cells by negatively regulating miR‐198 to promote CDK4 activity. Circ_0025039 depletion significantly reduced glucose consumption rate and inhibited CM cell proliferation and invasion [142]. Circ_0084043 expression was abnormally enhanced in *BRAF*‐mutant CM cells, as above reported. Of interest, circ_0084043 could also contribute to glycolysis in A375 and A378 cells via the modulation of the miR‐31/KLF3 axis [143]. In a similar way, the lncRNA H19 sponged miR‐106a‐5p to upregulate E2F3 expression and consequently enhanced glucose metabolism in A375 cells. Notably, both KLF3 and E2F3 participated with the lncRNA NEAT1 to form a regulatory axis that promoted *BRAF*‐mutant CM cell proliferation, migration, and invasion [144]. These data clearly confirm that circRNAs and lncRNAs closely cooperate to regulate *BRAF*‐mutant CM through different pathways.

Besides regulating the miR‐625/YY1 axis, circ_0016418 acted as a decoy for miR‐605‐5p which directly bound to glutaminase, the rate‐limiting enzyme in glutamine metabolism [145]. Consequently, circ_0016418 depletion impeded glutamine catabolism in A375 and A875 cells and impeded tumor progression. Similarly, the lncRNA *OIP5* antisense RNA (OIP5‐AS1) sponged miR‐217 to upregulate glutaminase expression, thus promoting glutamine catabolism in SK‐MEL‐1 and SK‐MEL‐5 [146]. A switch from glucose to glutamine metabolism and an enhanced dependence on glutamine over glucose for cell proliferation is usually observed in BRAFi‐resistant CM [147]. Hence, these data provide valuable insights for future research, which may be directed to evaluate relationship between ncRNAs, glutamine metabolism, and response to targeted therapy in *BRAF*‐mutant CM.

## 5 CircRNAs and lncRNAs in CM immune regulation

**Fig. 4:**
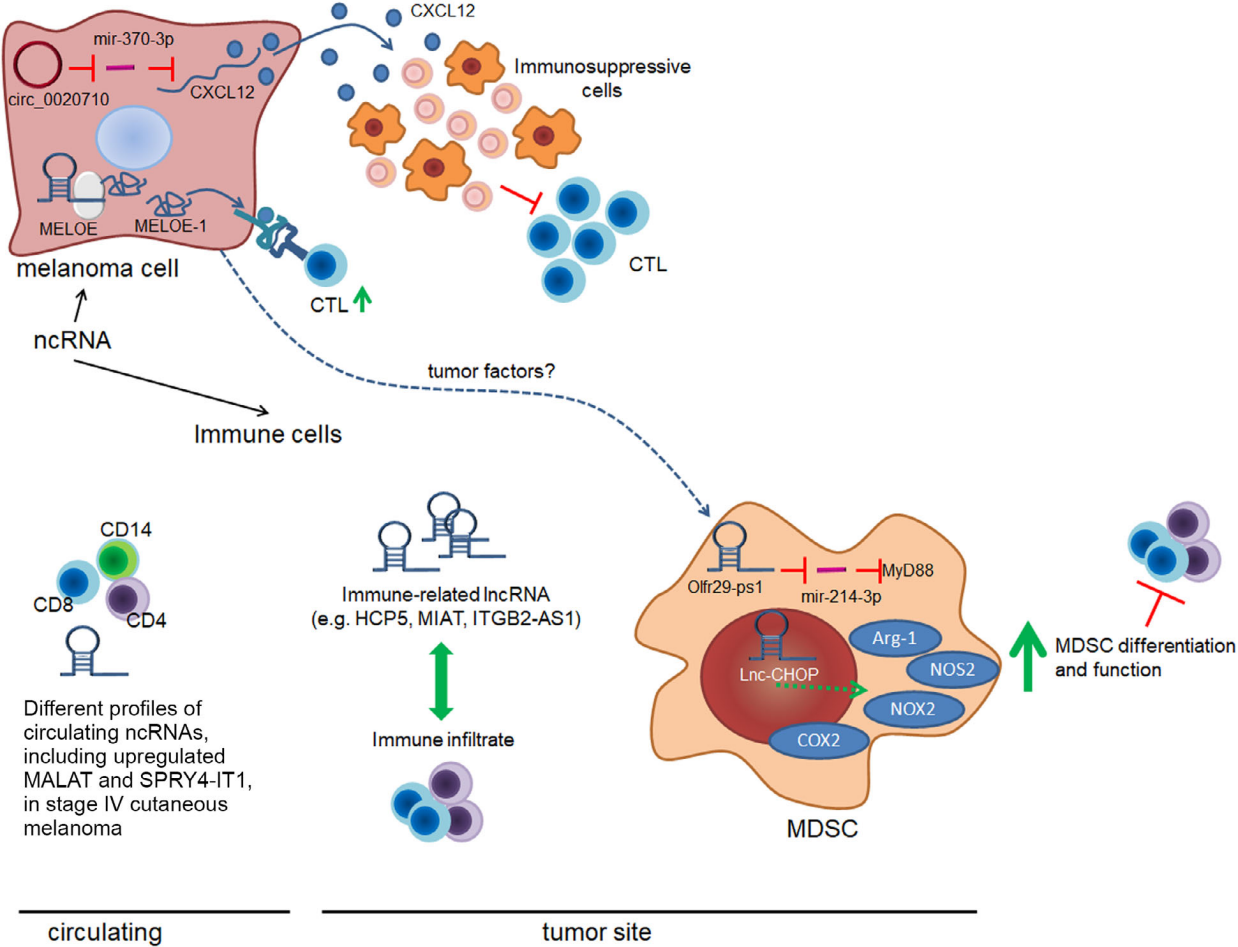
Roles of ncRNAs in CM‐immune system interaction. NcRNAs can impact on immune cell differentiation, function, and interaction with CM by acting either in cancer cells or in immune cells. In CM cells, the expression of ncRNAs could be both immunosuppressive and immunostimulating. Indeed, an impaired CTL (cytotoxic lymphocyte) infiltration can be observed in tumors expressing circ_020710, whereas the translation of lncRNA MELOE into the MELOE‐1 protein can improve CM immunogenicity. Immune cells, as well, express plenty of lncRNA. The mechanistic activity of lncRNA was studied more in detail in myeloid‐derived suppressor cells (MDSCs), where Olfr29‐ps1 and Lnc‐CHOP, with the possible contribution of tumor factors, are involved in MDSC differentiation and function. In line with the role of lncRNA in immune cell functions and with notion that the immune system is altered in cancer, CD4, CD8, and CD14 circulating cells from patients with stage IV CM were demonstrated to have different lncRNA profiles than those in healthy people. Green arrows and blocking bars indicate, respectively, the positive or negative regulation.

At present, little is known about the effects of circRNAs on immune regulation in CM. However, it has become recently clear that their targeting may have therapeutic potential for overcoming immunotherapy resistance. This is supported by the study of Wei CY *et al*., who focused on circ_0020710, that derives from the *CD151* gene. Besides promoting CM cell proliferation, migration, and invasion both *in vitro* and *in vivo*, elevated circ_0020710 levels could favor tumor immune escape. Mechanistically, circ_0020710 sponged miR‐370‐3p to protect CXCL12 from downregulation, thus creating an immunosuppression microenvironment that finally led to the exhaustion of cytotoxic T lymphocytes (CTL). Interestingly, the use of a CXCL12‐specific siRNA or the CXCL12 inhibitor AMD3100 reduced the circ_0020710‐induced malignant phenotype of CM cells. More importantly, treatment with AMD3100 and anti‐PD‐1 significantly attenuated *in vivo* tumor growth, indicating that the inhibition of circ_0020710/CXCL12 increased CTL infiltration and restored the efficacy of anti‐PD‐1 immunotherapy [148] (Fig. 4).

On the other hand, no data regarding lncRNAs and immune checkpoint inhibitors relationship are available in the literature. However, lncRNAs might play vital roles in immunotherapy resistance, since they are likely to control the homeostasis and functions of immune cells in CM (Fig. 4). In fact, RNA sequencing (RNA‐seq) analysis in diverse immune cell types (i.e., CD4^+^, CD8^+^, and CD14^+^ cells) identified a differential lncRNA expression profile between healthy subjects and stage IV CM patients, which usually develop resistance upon immunotherapy treatment. Functional enrichment analysis revealed that these lncRNAs were associated with several immune‐related and the PD‐1 checkpoint pathways. Metastasis‐associated lung adenocarcinoma transcript 1 (MALAT1) and SPRY4‐IT1 expression was also detected in stage IV CM patients and showed differential expression patterns between healthy subjects and patients with stage IV melanoma and in each of the three cell types [149]. Interestingly, MALAT1 was recently found to positively regulate PD‐L1 in non‐small‐cell lung cancer [150] and diffuse large B‐cell lymphoma (DLBCL) [151]; furthermore, MALAT1 expression promoted DLBCL immune escape by regulating the proliferation and apoptosis of CD8^+^ T cells. This evidence would support future research aimed at exploring the role of MALAT1 in regulating immune cell function and immune response in CM. A more recent analysis found that among lncRNAs whose expression was correlated with immunology in CM, 56% were significantly associated with CD8^+^ T‐cell infiltration in CM [152], which has been demonstrated to be a useful biomarker to predict prognosis and response to therapy in CM patients [153]. Intriguingly, some of these lncRNAs have already been demonstrated to participate in immune regulation. In particular, the *integrin subunit beta 2* antisense RNA 1 (ITGB2‐AS1) was found to be involved in the regulation of T‐cell and B‐cell activation [154], whereas the HLA class I histocompatibility antigen protein P5 (HCP5) is known for its functional roles in adaptive and innate immune responses [155]. Hence, besides controlling the miR‐1286/RARRP3 axis [156], HCP5 might also regulate CM immunogenicity. The myocardial infarction‐associated transcript (MIAT) was another lncRNAs which expression was significantly associated with the infiltration of immune cells in CM [152, 157]. Notably, although MIAT expression promoted CM cell proliferation, invasion, and migration [158], in a study of Liu *et al*. [157] CM patients with high expression of MIAT carried out a better prognosis, raising questions about its function in the control of immune response in CM.

Interestingly, lncRNAs were also shown to improve antigen presentation in CM (Fig. 4). For example, MELOE RNA represents a polycistronic lncRNA which is translated into MELOE‐1, MELOE‐2, and MELOE‐3 by different translational approaches: MELOE‐1 and MELOE‐2 are translated by an alternative internal ribosome entry sequence‐dependent mechanism exclusively in CM cell lines while MELOE‐3 is translated in a cap‐dependent manner, both in melanocytes and in CM cell lines [159, 160, 161]. In vitro experiments revealed a very scarce MELOE‐3‐specific T‐cell repertoire as compared to MELOE‐1 which could be recognized by tumor‐infiltrating lymphocytes and displayed the highest immunogenicity [159]. Based on these data, MELOE‐1 antigen is currently exploited as an immunotherapeutic target in a T‐cell immunotherapy clinical trial to treat metastatic CM patients (NCT02424916). LncRNAs have also proven to be associated with immune evasion since they may regulate the recruitment and activity of immunosuppressive cells, such as myeloid‐derived suppressor cells (MDSCs) (Fig. 4). As published by Shang *et al*. [162], the lncRNA *olfactory receptor 29*, pseudogene 1 (Olfr29‐ps1) could sponge miR‐214‐3p to promote MDSC differentiation into monocytic MDSCs with higher suppressive activities. By using a murine B16 melanoma model, *in vivo* experiments further demonstrated that Olfr29‐ps1 knockdown on MDSC decreased their immunosuppressive function. Moreover, smaller tumor volume and lighter tumor weight were detected in mice injected with Olfr29‐ps1‐knockdown MDSCs, and an increased number of CD4+ and CD8+ T cells was found in the tumor tissues compared with the control group. On the other hand, the mice injected with Olfr29‐ps1‐overexpressing MDSCs exhibited faster tumor development, larger tumor volume, heavier tumor weight, and fewer CD4+ and CD8+ T cells respect to control mice [162]. Likewise, the intronic C/EBP homologous protein long noncoding RNA (lnc‐CHOP) positively regulated MDSC generation and promoted tumor growth in murine B16 melanoma model [163]. Mechanistically, as observed by Gao *et al*. [163], lnc‐CHOP bound to CHOP and liver‐enriched inhibitory protein to regulate a large set of target transcripts in MDSCs, thus promoting their differentiation and immunosuppressive function in inflammatory and tumor environments. Altogether, these data indicate that the targeting of these immune‐related lncRNAs might negatively regulate the immunosuppressive abilities of MDSCs, and possibly improve CM patient’s response to immunotherapy.

In summary, there is still a lack of research on how lncRNAs regulate the function of tumor immune cells; therefore, further investigation in this field will be crucial to better elucidate the immune pathway regulation in CM in order to improve immunotherapy effectiveness.

## 6 Databases for the prediction and validation of circRNAs and lncRNAs

The last years have seen a rapid expansion in the number of bioinformatic resources for circRNA study, including circRNA identification algorithms, circRNA annotation databases and other tools implemented to create networks, or for visualization and computing their expression.

The two fundamental steps that allow circRNA identification are represented by the RNA library construction and sequencing. The RNase R treatment, polyadenylation, and poly(A)+ RNA depletion (RPAD) method enable the isolation of highly pure circRNA [164], whereas the RNA‐seq of RPAD‐isolated RNA analysis can be used to uncover new circRNAs. However, other library preparation strategies can be applied for circRNA identification [165], with a variable specificity in their detection. In addition, paired‐end sequencing method is also preferred to single end, improving the discovery of back‐spliced junction (BSJ) reads, that represent a molecular signature to detect circRNAs [166]. Most of the tools implemented for the identification of circRNA are stand‐alone and perform a remapping of the sequenced reads. A list of representative circRNAs identification tools is shown in Table 2. Among them, Find_circ [29], CIRI [167], and CIRCexplorer [168] use raw RNA‐seq reads, while DCC [169] employs the output of STAR aligner to detect BJS reads. Other tools largely used for circRNA identification, and based on BJS reads, are KNIFE [170], segemehl [171], Ularcirc [172], and UROBORUS [173]. Recently, machine learning approaches have also been applied to predict circRNAs, using several models classified on their known features (i.e., the conservation of transposable element, tandem repeats, open reading frame length, and single nucleotide polymorphism density). These tools mainly include DeepCirCode [174], PredcircRNA [175], WebCircRNA [176], and PredicircRNATool [177]. It is noteworthy that integration of different circRNA identification tools can reduce the false‐positive rate [178, 179, 180]. Users can combine or compare the results of different circRNA prediction tools to improve sensitivity and specificity of circRNA identification. Most of these pipelines are implemented in Python, Perl, or R and run in Linux or Unix‐like system. Although these tools are well‐documented with tutorials to help users, some computer science skills may be needed to perform an analysis. Therefore, a stand‐alone tool with a user‐friendly interface or a web‐tool could help users without advanced computational training. Of note, a comprehensive overview and evaluation of the circRNA detection tools have recently been described by Zeng and colleagues [181] and Chen and colleagues [182]. The quantification of circRNA expression is another important step in studying this class of ncRNAs. Generally, it is performed from the tools designed to identify them, and it is determined computing the ratio between back‐spliced junction reads and normal splicing junction reads, named circular‐to‐linear ratio. It represents the ratio of circRNA and linear RNA to obtain an overall expression value [183]. However, other strategies have been implemented, such as the one applied in Sailfish‐circ tool (https://github.com/zerodel/sailfish‐cir) [184] that quantifies circRNA abundance by transforming circRNA to pseudolinear transcript.

**Table 2:** Selected circRNA identification tools. The column “Category” describes the type of the tool. “Annotation” label indicates tool using a gene annotation file; otherwise, it is labeled with “*De novo*.”

Name	Last update	Category	Link	Reference
CIRCexplorer	2019	*De novo*; annotation	https://github.com/YangLab/CIRCexplorer2	[168]
CIRI	2017	*De novo*	https://sourceforge.net/projects/ciri/	[167]
DCC	2019	Annotation	https://github.com/dieterich‐lab/	[169]
DeepCirCode	2019	*De novo*; annotation	https://github.com/BioDataLearning/DeepCirCode	[174]
Find_circ	2015	*De novo*	https://github.com/marvin‐jens/find_circ	[29]
KNIFE	2016	Annotation	https://github.com/lindaszabo/KNIFE	[170]
PredcircRNA	2017	*De novo*; annotation	https://github.com/xypan1232/PredcircRNA	[175]
PredicircRNA Tool	2016	Annotation	https://sourceforge.net/projects/predicircrnatool/files/	[177]
Segemehl	2018	Annotation	https://www.bioinf.uni‐leipzig.de/Software/segemehl/	[171]
Ularcirc	2019	Annotation	https://github.com/VCCRI/Ularcirc	[172]
UROBORUS	2018	Annotation	https://github.com/WGLab/UROBORUS	[173]
WebCircRNA	2018	*De novo*; annotation	https://rth.dk/resources/webcircrna/	[176]

At present, several circRNA databases have been established, all containing a large number of circRNAs (Table 3) [summarized in [185]]. For instance, circBase [186] annotates circRNAs based on data from nine published papers, and for each circRNA reports several types of information, such as the sequence and the genomic coordinates. CircFunBase [187] and CIRCpedia [188] also represent useful tools that resume circRNA expression profiles and annotation from six species with data from different cell types or tissues. Other databases of note are CircRNADb [189], which contains information on circRNA with protein‐coding potential, CircInteractome [190, 191], that includes interaction of circRNAs with other ncRNAs as well as expression data, and CircNet [192], that integrates miRNA‐target networks, genomic annotation, expression profiles, and circRNA sequences. Due to clinical implication of circRNAs, some databases link circRNAs and diseases. For example, circ2Traits [193] lists 1951 human circRNAs potentially associated with 105 different diseases and details miRNA–circRNA–mRNA–lncRNA interaction network for each of these diseases. The main problem in circRNA databases is given by the nomenclature. To date, there is no unified nomenclature for circRNAs, and IDs used in the different databases are not universal. A standard unified nomenclature would facilitate data integration from different databases.

**Table 3:** Selected circRNA databases.

Database	Year	Annotation tool	Link	Reference
Circ2Disease	2018	Manually curated	http://bioinformatics.zju.edu.cn/Circ2Disease/index.html	[217]
Circ2Traits	2019	NA	https://github.com/shaoli86/circ2Traits	[193]
Circbase	2017	Manually curated	http://www.circbase.org/	[186]
CircFunBase	2019	Manually curated	http://bis.zju.edu.cn/CircFunBase/index.php	[187]
Circinteractome	2018	circBase	https://circinteractome.nia.nih.gov/	[190, 191]
CircNet	2016	Manually curated	http://circnet.mbc.nctu.edu.tw/	[192]
Circpedia	2018	CIRCexplorer2	http://www.picb.ac.cn/rnomics/circpedia	[188]
CircR2Disease	2018	Manually curated	http://bioinfo.snnu.edu.cn/CircR2Disease/	[218]
CircRNADb	2016	Manually curated	http://202.195.183.4:8000/circrnadb/circRNADb.php	[189]
CircRNADisease	2018	Manually curated	http://cgga.org.cn:9091/circRNADisease/	[219]

LncRNA association with other regulatory RNAs and proteins can be computationally determined using several approaches, previously used to predict miRNA or transcription factor targets. These strategies are generally based on the identification of functional similarity patterns extracted from sequences, of gene co‐expression, and of evolutionary conservation relationships [194]. Machine learning approaches have also been applied to predict RNA–RNA or RNA–protein interaction, starting from a large collection of known lncRNA–RNA interactions [195]. In view of the increasing interest in lncRNAs, several databases comprising experimentally validated and computationally predicted lncRNA interactions have recently been developed. For instance, STARBase deciphers protein–RNA and miRNA‐target interactions, thus allowing to decode lncRNA/miRNA/mRNA interaction networks [196]. Other databases of interest are listed in Table 4.

**Table 4:** Selected lncRNA databases.

Database	Year	Link	Reference
ChIPBase	2016	http://rna.sysu.edu.cn/chipbase/	[220]
LncBase	2016	https://carolina.imis.athena‐innovation.gr/diana_tools/web/index.php?r=lncbasev2%2Findex‐experimental	[221]
LNCipedia	2019	https://lncipedia.org/	[222]
LncRNAdb	2010	https://rnacentral.org/expert‐database/lncrnadb	[223]
LncRNADisease	2019	http://www.cuilab.cn/lncrnadisease	[224]
LncRNome	2012	http://genome.igib.res.in/lncRNome/	[225]
miRNet	2020	https://www.mirnet.ca/miRNet/home.xhtml	[226]
Noncode v6.0	2017	http://www.noncode.org/	[227]
STARBase	2013	http://starbase.sysu.edu.cn/starbase2/index.php	[196]

## 7 Conclusions

Recently, circRNAs and lncRNAs have attracted intensive interest due to their potential functions in CM biology. These ncRNAs have often pleiotropic effects by which they can affect different pathways rather than acting predominantly through a specific target gene. Therefore, by functioning as regulators of gene expression, they contribute to increase the growth and spread of CM cancer cells, making them valuable biomarkers and ideal therapeutic targets. Classical circRNA and lncRNA targeting involves the use of RNA interference approaches, whereas ASO technology can be employed to ablate lncRNA expression. Considering that circRNAs and lncRNAs could be located in the nucleus [197], genome editing using CRISPR/Cas‐9 system could also serve as an intriguing method to trigger their silencing [198] [summarized in [199]]; however, additional research is needed for its eventual application in the clinic. An alternative approach to target circRNA and lncRNA interactions would be the use of small‐molecule inhibitors that can disrupt lncRNA secondary structure or inhibit their association with miRNAs. Despite these findings, at present, circRNA and lncRNA therapeutic targeting remains mainly at the laboratory stage.

Although a large number of studies have indicated ncRNA deregulation in *BRAF^V600^*‐mutant CM, only a number of papers are about the role of lncRNAs in response to targeted therapies, whereas no information on circRNA involvement in BRAFi/MEKi resistance is available. Similarly, research on the role of circRNAs and lncRNAs in the resistance of CM to immunotherapy is still at the nascent stage. Therefore, there are many unknown questions about circRNAs and lncRNAs that need to be further explored in CM.

## Conflict of interest

MM has served as a consultant and/or advisor to Roche, Bristol‐Myers Squibb, Merck Sharp Dohme, Incyte, AstraZeneca, Amgen, Pierre Fabre, Eli Lilly, Glaxo Smith Kline, SciClone, Sanofi, Alfasigma, and Merck Serono; MM and AC own shares in Epigen Therapeutics, SRL. The remaining authors declare that the research was conducted in the absence of any commercial or financial relationships that could be construed as a potential conflict of interest.

## Author contributions

BM and EF wrote the initial manuscript and prepared the tables. GG, GP, LS, and EF designed the figures. All authors contributed to writing and finalized the manuscript. All authors read and approved the final manuscript.

## Supporting information


**Table S1**. Summary of circRNAs and lncRNAs discussed in the text.
