# Supplementary material for: The pleiotropic roles of circular and long noncoding RNAs in cutaneous melanoma
Source: Mol Oncol. 2021 Jun 18;16(3):565–93. doi: 10.1002/1878-0261.13034 (PMC8807361; doi:10.1002/1878-0261.13034)
Supplement: Supplementary file 1 — Table S1. Summary of circRNAs and lncRNAs discussed in the text. [file MOL2-16-565-s001.doc]

**Supplementary Table 1. Summary of circRNAs and lncRNAs discussed in the text.**

| **CircRNA ID** | **Spliced sequence lenght (base pairs)** | **Locus** |
| --- | --- | --- |
| CDR1as | 1485 | chrX:139865339-139866824 |
| circ_0000082 | 319 | chr1:64515362-64516388 |
| circ_0008157 | 671 | chr11:118425173-118430579 |
| circ_0016418 | 719 | chr1:213134507-213147412 |
| circ_0020710 | 1545 | chr11:832951-838831 |
| circ_0023988 | 476 | chr11:89155069-89185063 |
| circ_0025039 | 321 | chr12:2975558-2977920 |
| circ_0030388 | 442 | chr13:64564228-64608670 |
| circ_0084043 | 1452 | chr8:38883321-38959449 |
| circ_ITCH | 873 | chr20:33001547-33037285 |
| circANRIL | n.a. | 9p21 |
| circMYC | 555 | chr8:128748314-128748869 |
| **LncRNA ID** | **Lenght (base pairs)** | **Locus** |
| ANRIL | 3834 | 9p2 |
| ATB | 2446 | 14q11.2 |
| BANCR | 693 | 9q21.11-q21.12 |
| CASC2 | 3285 | 10q26 |
| CPS1-IT1 | 2306 | 2q34 |
| CRNDE | 8903 | 16q12.2 |
| DIRC3 | 3384 | 2q35 |
| FENDRR | 3606 | 16q24.1 |
| FOXD3-AS1 | 3892 | 1p31.3 |
| GAS5 | 650 | 1q25 |
| GAS6-AS2 | 1942 | 13q34 |
| H19 | 2300 | 11p15.5 |
| HCP5 | 2547 | 6p21.33 |
| HOTAIR | 2158 | 12q13.13 |
| ITGB2-AS1 | 2425 | 21q22.3 |
| KCNQ1OT1 | 91671 | 11p15.5 |
| LINC00173 | 1597 | 12q24.22 |
| LINC00518 | 3018 | 6p24.3 |
| LINC00632 | 1398 | Xq27.1 |
| LINC00961 | 1546 | 9p13.3 |
| LINC00963 | 9557 | 9q34.11 |
| LINC-PINT | 1430 | 7q32.3 |
| Lnc-CHOP (murine) | ~1800 | 11 |
| MALAT1 | 8708 | 11q13 |
| MEG3 | 9689 | 14q32.2 |
| MELOE | n.a | n.a |
| MHENCR | 795 | 20q13.33 |
| MIAT | 8760 | 22q12.1 |
| MIR31HG | 2166 | 9p21.3 |
| MIR4435-2HG | 2968 | n.a. |
| MIRAT | n.a. | 8q.24.12 |
| NEAT1 | 3756 | 11q13.1 |
| Olfr29-ps1 (murine) | 963 | 4 |
| OIP5‐AS1 | 8865 | 15q15.1 |
| Orilnc1 | n.a. | n.a. |
| OVAAL | 1489 | 1q25.3 |
| PEG10 | 763 | 7q21 |
| PVT1 | 1957 | 8q24.21 |
| RMEL3 | 3791 | 5q11.2 |
| SAMMSON | 2166 | 3p13-3p14 |
| SLNCR1 | 2257 | 17q24.3 |
| SPRY4-IT1 | 703 | 5q31.3 |
| SSATX | 1195 | Xp22.1 |
| SRA | 687 | 5q31.3 |
| TUG1 | 7598 | 22q12.2 |
| UCA1 | 1456 | 19p13.12 |
| ZEB1-AS1 | 2449 | 10p11.22 |

^*^data not available
